# Hypoadrenocorticism‐like syndrome in a cat with *Tritrichomonas foetus* infection: a case report

**DOI:** 10.1111/jsap.70091

**Published:** 2026-02-08

**Authors:** M. Crisonà, F. Fracassi, A. M. Tardo, S. Okonji, F. Del Baldo

**Affiliations:** ^1^ Department of Veterinary Medical Sciences University of Bologna Ozzano dell’Emilia Italy

## Abstract

A reduced sodium: potassium ratio is an uncommon finding in cats, and is typically associated with conditions such as hypoadrenocorticism, severe renal and cardiovascular diseases, cavitary effusion and certain non‐parasitic gastrointestinal diseases. This report describes a case of a 1‐year‐old female Maine Coon cat showing severe hyperkalaemia and hyponatraemia, associated with gastrointestinal signs and *Tritrichomonas foetus* infection. Adrenocorticotropic Hormone (ACTH) stimulation testing excluded hypoadrenocorticism. Supportive care combined with antiprotozoal treatment using ronidazole led to long‐term clinical resolution and normalisation of the electrolyte abnormalities.

## INTRODUCTION

A reduced sodium:potassium (Na^+^:K^+^) ratio is an uncommon finding in cats (Bell et al., 2005). It is a hallmark clinical‐pathological finding of primary typical hypoadrenocorticism and reflects aldosterone deficiency (Feldman et al., 2014, 2019; Roberts et al., 2025; Sieber‐Ruckstuhl et al., 2023). Nevertheless, comparable electrolyte patterns may develop in non‐adrenal disorders despite normal to increased plasma aldosterone concentrations (Graves et al., 1994; Willard et al., 1987), such as renal and urinary tract diseases (DiBartola & De Morais, 2012b; Marino & Foster, 2024), cavitary effusions (Bissett et al., 2001; Thompson & Carr, 2002; Willard et al., 1991), gastrointestinal non‐parasitic diseases in cats (Baker et al., 2024; Bell et al., 2005), parasitic diseases in dogs (Car et al., 2019; DiBartola et al., 1985; Kitchell et al., 2000; Malik et al., 1990; Ruckstuhl et al., 2002; Venco et al., 2011), non‐adrenal endocrine diseases (Bell et al., 2005), neoplastic diseases (Lamb & Muir, 1994), cardiovascular diseases (Bell et al., 2005; Nielsen et al., 2008) and late pregnancy in dogs (Schaer et al., 2001). Clinical signs in some of these disorders may mimic hypoadrenocorticism. A previous investigation of cats with reduced Na^+^:K^+^ ratios identified a variety of aetiologies, including non‐parasitic gastrointestinal disease (25% of cats), pseudohyperkalaemia as a consequence of lipaemia or EDTA contamination of the samples (12.2% of cats), urinary diseases, diabetes mellitus, hyperthyroidism, cardiovascular diseases and cavitary effusions. However, no cases secondary to parasitic intestinal disease were identified (Bell et al., 2005).

Electrolyte patterns suggestive of primary hypoadrenocorticism (termed Pseudo‐Addison) have also been documented in dogs with primary gastrointestinal diseases (Car et al., 2019; DiBartola et al., 1985; Graves et al., 1994; Malik et al., 1990; Ruckstuhl et al., 2002; Venco et al., 2011). Trichyuriasis has been reported as the main underlying disorder (Car et al., 2019, DiBartola et al., 1985, Graves et al., 1994, Malik et al., 1990, Ruckstuhl et al., 2002, Venco et al., 2011), but other conditions have included concurrent ancylostomiasis (DiBartola et al., 1985), ascariasis (Roth & Tyler, 1999), enteric salmonellosis, gastric torsion and perforating duodenal ulcers (DiBartola et al., 1985). It is supposed that a key role in the pathogenesis of electrolytic abnormalities in dogs with gastrointestinal parasitic and non‐parasitic diseases is played by the gastrointestinal electrolytic and fluid losses, leading to dehydration and hypovolaemia (Bell et al., 2005; DiBartola & De Morais, 2012b). In contrast to what has been reported in dogs, there are no reports of parasitic diseases causing a low Na^+^:K^+^ ratio in cats. This case report is the first to describe a cat with hypoadrenocorticism‐like syndrome and concurrent *Tritrichomonas foetus* infection, diagnosed on faecal examination. With the term hypoadrenocorticism‐like syndrome, the authors refer to the electrolytic derangement with hyperkalaemia and hyponatraemia associated with gastrointestinal signs, which resemble hypoadrenocorticism.

## CASE DESCRIPTION

A 1‐year and 6‐month‐old, 3.5 kg entire female Maine Coon cat was presented to the Veterinary Teaching Hospital of the University of Bologna for diarrhoea for at least 4 months of duration and weight loss. She was a breeding female, living in a multi‐cat household and some of the cohabitants also showed diarrhoea. She was regularly vaccinated; deworming was not regular. The cat showed both large and small bowel signs, characterised by liquid mucoid diarrhoea (faecal score 6 to 7 based on Purina Fecal Scoring Chart) with increased volume of faeces, increased frequency of defecation, presence of mucus, urgency and weight loss. The cat also had a history of a reduced appetite and mental dullness a few days before the visit. The physical examination showed a body condition score (BCS) of 3/9, mental dullness with lateral recumbency, severe dehydration (10%), hypothermia (rectal temperature of 36.7°C), heart rate of 190 bpm and systolic blood pressure of 110 mmHg (SunTech® Vet30 oscillometric blood pressure monitor). The cat was considered to be in a state of compensated shock. Venous blood gas analysis, complete blood count, serum chemistry analysis, urinalysis and abdominal ultrasonography were performed. Venous blood gas analysis, performed by ABL 825 FLEX blood gas analyser, revealed mild metabolic and respiratory alkalosis (pH 7.43, reference interval (RI) 7.34 to 7.40; HCO_3_^−^ 25.9 mmol/L, RI 18.0 to 23.2 mmol/L; pCO_2_ 39.9 mmHg, RI 40 to 60 mmHg), moderate hyperkalaemia (6.7 mmol/L, RI 3.3 to 5.5 mmol/L) and severe hyponatraemia (107 mmol/L, RI 139 to 151 mmol/L) with a Na^+^:K^+^ ratio of 16 (normal >27). Additional abnormalities included hypochloraemia (81 mmoL/L, RI 110 to 122 mmol/L), ionised hypocalcaemia (1.17 mmol/L, RI 1.20 to 1.41 mmol/L) and mild elevation in lactate (2.9, normal <2.87).

Haematology showed mild leucocytosis (17.5 × 10^3^/μL, RI 4.8 to 14.93 × 10^3^/μL) and neutrophilia (15,710/μL, RI 1600 to 10,000/μL). Serum chemistry abnormalities are shown in Table 2. Urinalysis showed USG 1.019 and proteinuria (urinary protein/creatinine ratio 1.1, RI 0.2 to 0.4).

Abdominal ultrasound revealed a diffuse intestinal wall thickening, most pronounced in the colon, with decreased peristaltic movements, significant distention of both stomach and large bowel with fluid and mild jejunal lymphoadenomegaly. The adrenal glands appeared normal in both size and morphology.

ACTH (Synachten, Alfasigma S.p.A.; 125 μg IV) stimulation test was performed. Baseline and 1‐hour post‐ACTH serum cortisol concentrations, measured using the Siemens Immulite® 2000 XPI Immunoassay System, were 176.6 and 303.6 nmol/L (normal >55 nmol/L) respectively, ruling out hypoadrenocorticism.

Standard three‐lead electrocardiography did not show any abnormalities.

The cat was treated with two boluses of 5 mL/kg and a bolo of 10 mL/kg over 20 minutes of crystalloid fluid (Ringer’s lactate solution, [Na^+^]: 132 mEq/L), with repeat assessment after each bolus. After fluid resuscitation, it was set a replacement rate of 6 mL/kg/h of Ringer’s lactate solution, then tapered to 4 mL/kg/h during the first 24 hours of hospitalisation. In the following days, the type and rate of fluids were adjusted according to the ongoing fluid losses and sodium concentration. Supportive therapies with a high‐digestible diet (Royal Canin Gastroinstestinal), maropitant (Cerenia Zoetis Italia S.r.l.; 1 mg/kg IV q24h), metoclopramide (Vomend Dechra Veterinary Products S.r.l.; 1 mg/kg/day IV CRI) and probiotics (Nucron Aurora Biofarma; Enterina BuonaPet OS q12h) were also instituted. Whole blood electrolyte concentrations were monitored at 3‐, 18‐ and 24‐hour post‐admission. At 18 hours, potassium concentration was within reference interval. Hyponatraemia persisted, although sodium concentration had increased (129 mmol/L; 22 mmol/L over 18 hours; 1.2 mmol/L per hour). Other laboratory data during hospitalisation are shown in Tables 1 and 2. The body temperature normalised and cat’s mental status and appetite improved over 24 hours post‐admission. However, the cat had persistent liquid diarrhoea and urgency, with consequent faecal incontinence.

**Table 1:** Venous blood gas analysis at first presentation, during hospitalisation and after discharge

Parameters	T0	10 minutes*	3 hours*	18 hours*	24 hours*	30 hours*	48 hours*	72 hours*	1 month*	Reference interval
pH	7.41	7.32	7.41	7.43	7.42	7.36	7.39	7.34	7.32	7.34 to 7.40
HCO_3_^−^ (mmol/L)**	23.9	24.4	23.7	30.3	27.1	18.6	24.5	26.5	19.8	18.0 to 23.2
pCO_2_ (mmHg)	39.9	48.5	38.1	46.5	43.3	34.1	42.4	52.6	39.9	40 to 60
K^+^ (mmol/L)	6.7	7.1	6.9	3.7	3.8	3.7	3.1	3.7	3.8	3.3 to 5.5
Na^+^ (mmol/L)	107	107	111	129	128	130	137	143	148	139 to 151
Ca^++^ (mmol/L)	1.17	1.16	1.22	1.17	1.25	1.29	1.20	1.23	1.31	1.20 to 1.41
Cl^−^ (mmol/L)	81	97	93	101	102	106	111	128	126	110 to 122
Anion gap (mmol/L)	7.5	10.8	0.9	1.4	2.4	9	4.1	−7.7	6.5	13 to 27***
Lactate (mmol/L)	2.9	5.3	2.2	2.2	3.3	1.4	0.7	0.8	0.8	<2.87
Na^+^/ K^+^	16.0	15.1	16.1	34.9	33.7	35.1	44.2	38.6	38.9	>27

* The time points refer to the admission (T0)

** HCO_3_^−^ is calculated by the analyser

*** See DiBartola (2012)

**Table 2:** Clinicopathological findings at presentation and during hospitalisation

Parameters	T0	24 hours	Reference range
Haematocrit (%)	45.3		32.0 to 48.0
CK (U/L)	734		91 to 326
LDH (U/L)	564		63 to 193
AST (U/L)	89		9 to 40
ALT (U/L)	86	62	20 to 72
SAP (U/L)	8		20 to 140
GGT (U/L)	0.1		0 to 4
Total bilirubin (U/L)	0.05	0.21	0 to 0.35
Cholesterol (mg/dL)	152		59 to 230
Triglycerides (mg/dL)	208		10 to 100
Fasting bile acids (mmol/L)	3.1		0 to 7.6
Glucose (mg/dL)	225		65 to 148
Fructosamine (μmol/L)	393.0		220 to 350
Total protein (g/dL)	9.69	7.55	6.5 to 8.8
Albumin (g/dL)	4.06	3.38	2.60 to 4.00
Globulin (g/dL)	5.63	4.17	
A/G ratio	0.72	0.81	0.52 to 1.20
Urea (mg/dL)	267		30 to 65
Creatinine (mg/dL)	1.98	0.83	0.80 to 1.80
Phosphorus (mg/dL)	8.14		2.5 to 6.2
SAA (μg/dL)	9		0 to 5
Cobalamin (ng/L)	367		250 to 1000

CK Creatine kinase, LDH Lactate dehydrogenases, ALT Alanine aminotransferase, AST Aspartate aminotransferase, ALP Alkaline phosphatase, GGT Gamma glutamyl transferase; A/G Albumin/globulin, SAA Seroamiloid A

**FIG. 1:**
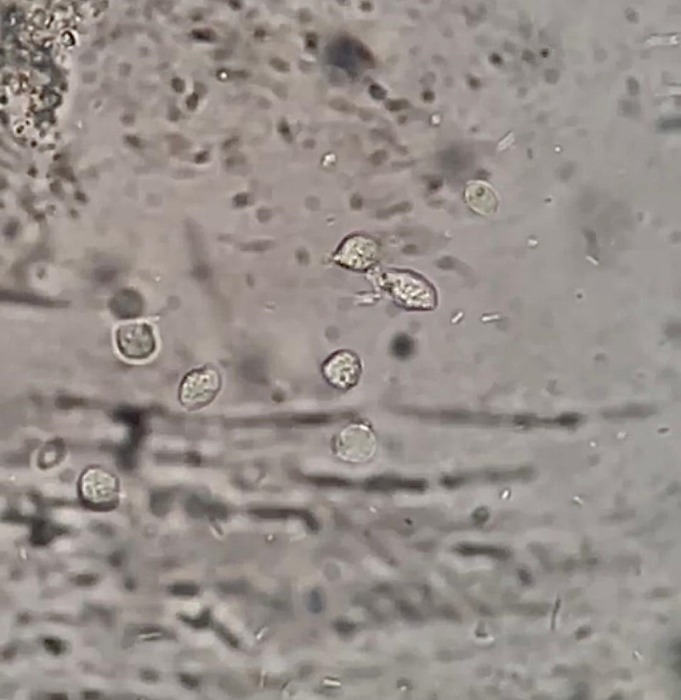
Microscopic visualisation of *Tritrichomonas foetus* trophozoites from a fresh faecal smear at 100× magnification. The faecal smear was prepared by placing faecal material on a laboratory slide along with saline solution.

Faecal examination on three faecal samples over 2 days was performed, including searching for *Giardia* spp. by merthiolate‐iodine‐formalin method. The faecal flotation was negative, while direct microscopic evaluation of a fresh faecal smear showed motile trophozoites consistent with *Tritrichomonas foetus* infection (Fig. 1 and Video S1).

On day 4 post‐admission, generalised seizure episodes, blindness and vocalisations were noted. Neurological examination showed mental dullness, confirmed blindness and absence of menace response. Based on the history of seizures and the results of the neurological examination, a forebrain neurolocalisation was suspected. The main differential diagnosis was osmotic demyelination syndrome (ODS), considering the rapid increase of blood sodium concentration (Burton & Hopper, 2019; Churcher et al., 1999; Harris et al., 2023; Lee et al., 2022), although thiamine deficiency, possibly secondary to intestinal malabsorption could not be entirely ruled out (Kritikos et al., 2017). Magnetic resonance imaging was proposed but it was declined by the owner. Anticonvulsive therapy was initiated, including levetiracetam (Keppra, UCB Pharma S.p.a.; 30 mg/kg OS q8h) and phenobarbital (Phenoleptil, Dechra Regulatory B.V.; 2 mg/kg OS q12h). Additional treatment included ronidazole (60 mg galenic preparation; 30 mg/kg PO q24h for 14 days), thiamine supplementation (Benerva, SF Group S.r.l; 20 mg/kg SC q12h) and, since serum cobalamin concentration was below 400 ng/L (Kather et al., 2020), cyanocobalamin supplementation (Epaviten, Teofarma; 50 mcg/kg SC q7d). No seizures were note after 24 hours of anticonvulsive therapy, while the mental status gradually improved over the next week. Faecal consistency progressively improved (Purina Fecal Scoring Chart, 5/9 on day 7 post‐admission). The cat was discharged 10 days post‐admission.

Follow‐up monitoring after 1 week, 2 weeks and 2 months revealed normalisation of faecal consistency (faecal score 2 to 3 based on Purina Fecal Scoring Chart) and weight gain (BCS 5/9, body weight 5.2 kg at the last follow‐up). Sodium and potassium concentrations normalised over 3 days of hospitalisation and were within their reference intervals after discharge. There was no recurrence of neurological signs and the antiepileptic drugs were gradually reduced until they were stopped within 2 months.

## DISCUSSION

This case report describes the development of hypoadrenocorticism‐like syndrome in a Main Coon cat with severe chronic gastrointestinal losses suspected to be secondary to *Tritrichomonas foetus* infection.

*Tritrichomonas foetus* infection was diagnosed by direct faecal microscopic examination. This parasite is a monocellular flagellate protozoon which typically resides in the ileum, cecum and colon of cats and it can cause severe local inflammation through its adhesion to the intestinal epithelium (Gruffydd‐Jones et al., 2013; Yao & Köster, 2015). Parasite adhesion is mediated by specific receptor–ligand interactions between *Tritrichomonas’* cell surface molecules and the intestinal cells, which causes cytotoxic effects by induction of apoptosis, resulting in deep injury of the colonic epithelium, sometimes extending to the ileum and distal jejunum (Tolbert & Gookin, 2016; Yao & Köster, 2015). Even though not all infections are associated with clinical signs (Gruffydd‐Jones et al., 2013; Yao & Köster, 2015), in some cats *Tritrichomonas foetus* infection can result in large bowel diarrhoea with mucus, haematochezia, proctitis and faecal incontinence; vomiting, anorexia and weight loss are also reported (Gookin et al., 1999; Gruffydd‐Jones et al., 2013; Yao & Köster, 2015), possibly leading to severe dehydration and hypovolaemia.

In the case here described, the main possible causes of electrolytic derangements have been ruled out; such as hypoadrenocorticism, oligo/anuric acute kidney injury, cavitary effusions and artefactual abnormalities. Electrolytic abnormalities were supposed to be linked to the severe gastrointestinal disease.

The pathogenesis of the electrolyte derangements in severe gastrointestinal diseases is not well understood; it is hypothesised that a key role could be played by the severe loss of isotonic or hypotonic fluids that occur in cats with vomitus and/or diarrhoea. It can cause depletion of ECF volume and a decline in glomerular filtration rate (GFR) and distal tubular flow rates. This causes activation of the renin‐angiotensin‐aldosterone system (RAAS) (to promote renal Na^+^ and water retention) and non‐osmotic stimulation of ADH release (to promote renal water retention). Although direct loss of Na^+^ in gastroenteric fluids or decreased Na^+^ intake due to inappetence or anorexia may contribute, hyponatraemia mainly results from dilution of ECF Na^+^ by retained water (DiBartola et al., 1985; DiBartola & De Morais, 2012a; Graves et al., 1994; Malik et al., 1990). Hyperkalaemia may develop if hypovolaemia and hyponatraemia persist. It is thought to be largely attributable to decreased renal excretion of K^+^ due to volume depletion and decreased distal tubular flow rates; low Na^+^ concentration in the lumen of the distal tubules can exacerbate hyperkalaemia by reducing the electrochemical gradient required for movement of K^+^ ions from tubular cells into the lumen (DiBartola & De Morais, 2012b). Metabolic acidosis arising from loss of bicarbonate (HCO_3_^−^) ions in diarrhoea may also promote hyperkalaemia via translocation of intracellular K^+^ into the ECF (DiBartola et al., 1985; DiBartola & De Morais, 2012b; Graves et al., 1994; Malik et al., 1990). The cat here reported did not showed metabolic acidosis, making the latter hypothesis unlikely. Another pathogenetic hypothesis on electrolytic abnormalities stay in the reduced sodium absorption and increased potassium release at a colonic level in cats with acute and chronic gastrointestinal signs, as a consequence of dysregulation of electrogenic sodium epithelial sodium channels (Baker et al., 2024). Concurrent hypocalcaemia and hypochloraemia are supposed to be due to the severe gastrointestinal losses, mild metabolic alkalosis can play a role in hypochloraemia (De Morais & Biondo, 2012), while vitamin D deficiency due to intestinal malabsorption as a cause of hypocalcaemia cannot be excluded (Baker et al., 2024; Schenck et al., 2012).

In dogs with a low Na^+^:K^+^ ratio associated with *Trichiuris vulpis* infection, the deep intestinal wall damage has also been hypothesised to play a role in the development of electrolytic abnormalities (DiBartola et al., 1985; Ruckstuhl et al., 2002; Venco et al., 2011). *Tritrichomonas foetus*, in a similar manner as *Trichiuris vulpis*, localises mainly in the colon of cats where it can cause severe local inflammation. Therefore, a similar pathogenesis in electrolytic derangements could be assumed. However, strong evidence on this mechanism is lacking. Moreover, in this case, electrolytic abnormalities normalised after rehydration before starting ronidazole treatment, this supports the hypothesis of severe dehydration as a key factor in the pathogenesis of hypoadrenocorticism‐like syndrome (Bell et al., 2005; Venco et al., 2011).

After the correction of electrolytic abnormalities and before the introduction of ronidazole treatment, the cat here described showed neurological signs characterised by severe mental depression and seizures. The most likely cause was considered to be the rapid correction of hyponatraemia, which can lead to a quick change in osmotic pressure and the so‐called ODS (Burton & Hopper, 2019; Churcher et al., 1999; Lee et al., 2022). Indeed, in the course of chronic hyponatraemia, translocation of electrolytes and organic osmolytes (such as glutamine, glutamate, taurine, myoinositol, phosphocreatine and glycerophosphorylcholine) from the ICF to the ECF lead to the equilibration of the osmotic gradient between these compartments. With rapid correction of sodium serum levels, the increased osmotic gradient lead to rapid translocation of water from the ICF to the ECF, with consequent fluid loss from brain cells, axons’ shrinkage and rupture of their connections with myelin sheats (Ambati et al., 2023; Churcher et al., 1999; Lee et al., 2022; Thurston et al., 1987). ODS has not been described previously in cats, nevertheless cases of non‐osmotic pontine demyelination due to suspected metabolic causes have been reported (Poncelet et al., 2011; Santifort et al., 2021). Moreover, ODS is a largely described phenomenon in humans (Ambati et al., 2023) and it was also previously described in dogs (Burton & Hopper, 2019; Churcher et al., 1999; Harris et al., 2023; Lee et al., 2022). In the case here reported, serum sodium levels increased by 22 mmol/L over the first 18 hours (1.2 mmol/L/h). To mitigate the risk of ODS, more intensive monitoring of sodium levels, along with appropriate adjustments to fluid therapy using hypotonic fluids, should have been instituted. Limitations of this case report include the inability to exclude viral causes of gastroenteritis, the absence of an intestinal biopsy to rule out inflammatory bowel disease and the potential for false‐negative results in faecal examinations. Consequently, we cannot exclude the possibility of other comorbidities that may have partially contributed to the electrolytic derangements and clinical signs observed in the cat.

In conclusion, this report describes a cat with a low Na^+^:K^+^ ratio, consistent with hypoadrenocorticism‐like syndrome secondary to *Tritrichomonas foetus* infection. This case underscores the importance of considering *Tritrichomonas foetus* as a differential diagnosis in cats presenting with electrolyte abnormalities and gastrointestinal signs. Moreover, this is also the first case of suspected ODS reported in cats, highlighting the importance of intensive monitoring in cats with severe hyponatraemia. Further studies are needed to understand the prevalence and pathophysiology of electrolyte abnormalities in cats with *Tritrichomonas foetus* infection and gastrointestinal signs.

### Author contributions

**M. Crisonà:** Writing – original draft preparation; formal analysis, data curation. **F. Fracassi:** Writing – review and editing. **A. M. Tardo:** Writing – review and editing. **S. Okonji:** Writing – review and editing. **F. Del Baldo:** Conceptualization; data curation; writing – review and editing; supervision.

### Conflict of interest

Drs Fracassi and Tardo are Associate Editors of the Journal of Small Animal Practice and co‐authors of this article. They were excluded from editorial decision‐making related to the acceptance of this article for publication in the journal.

## Supporting information


**Data S1.** Data files

**Video S1.** Microscopic visualisation of *Tritrichomonas foetus* trophozoites from a fresh faecal smear, to note the undulating membrane of the trophozoite

## Data Availability

The data that support the findings of this study are available from the corresponding author upon reasonable request.
