# Supplementary material for: Hypoadrenocorticism‐like syndrome in a cat with Tritrichomonas foetus infection: a case report
Source: J Small Anim Pract. 2026 Feb 8;67(6):569–73. doi: 10.1111/jsap.70091 (PMC13244411; doi:10.1111/jsap.70091)
Supplement: Supplementary file 1 — Data S1. Data files [file JSAP-67-569-s001.docx]

| **Table 1. Clinicopathological abnormalities at first presentation, during hospitalization and after discharge.** | | | | | | | | | | |
| --- | --- | --- | --- | --- | --- | --- | --- | --- | --- | --- |
| **Parameters** | **T0** | **10 min** | **3 hours** | **18 hours** | **24 hours** | **30 hours** | **48 hours** | **72 hours** | **3 weeks** | **Reference range** |
| pH | 7,41 | 7,32 | 7,41 | 7,43 | 7,42 | 7,36 | 7,39 | 7,34 | 7,32 | 7.34 … 7.40 |
| HCO_3_^-^ (mmol/l) | 23,9 | 24,4 | 23,7 | 30,3 | 27,1 | 18,6 | 24,5 | 26,5 | 19,8 | 18.0 … 23.2 |
| pCO_2_ (mmHg) | 39,9 | 48,5 | 38,1 | 46,5 | 43,3 | 34,1 | 42,4 | 52,6 | 39,9 | 32.7 … 44.7 |
| **K^+^ (mmol/l)** | **6,7** | **7,1** | **6,9** | **3,7** | **3,8** | **3,7** | **3,1** | **3,7** | **3,8** | **3.3 … 5.5** |
| **Na^+^ (mmol/l)** | **107** | **107** | **111** | **129** | **128** | **130** | **137** | **143** | **148** | **139 … 151** |
| Ca^++^ (mmol/l) | 1,17 | 1,16 | 1,22 | 1,17 | 1,25 | 1,29 | 1,20 | 1,23 | 1,31 | 1.20 … 1.41 |
| Cl^-^ (mmol/l) | 81 | 97 | 93 | 101 | 102 | 106 | 111 | 128 | 126 | 110 … 122 |
| CK (U/L) | 734 |  |  |  |  |  |  |  |  | 91 … 326 |
| LDH (U/L) | 564 |  |  |  |  |  |  |  |  | 63 … 193 |
| AST (U/L) | 89 |  |  |  |  |  |  |  |  | 9 … 40 |
| ALT (U/L) | 86 |  |  |  | 62 |  | 47 |  | 85 | 20 … 72 |
| Glucose (mg/dl) | 225 |  |  |  |  |  |  |  |  | 65 … 148 |
| DGGR lipase (U/L) | 113 |  |  |  |  |  |  |  |  | < 26 |
| Total protein (g/dl) | 9,69 |  |  |  | 7,55 |  | 6,99 |  | 7,12 | 6.5 … 8.8 |
| Albumin (g/dl) | 4,06 |  |  |  | 3,38 |  | 3,00 |  | 2,76 | 2.60 … 4.00 |
| Globulin (g/dl) | 5,63 |  |  |  | 4,17 |  | 3,99 |  | 4,36 |  |
| A/G ratio | 0,72 |  |  |  | 0,81 |  | 0,75 |  | 0,63 | 0.52 … 1.20 |
| Urea (mg/dl) | 267 |  |  |  |  |  |  |  |  | 30 … 65 |
| Creatinine (mg/dl) | 1,98 |  |  |  | 0,83 |  | 0,73 |  | 0,90 | 0.80 … 1.80 |
| P (mg/dl) | 8,14 |  |  |  |  |  |  |  |  | 2.5 … 6.2 |
| Na^+^/K^+^ ratio | 15,9 |  |  |  | 30 |  | 37 |  | 32 | > 27 |
| HCO_3_^-^ bicarbonate, pCO_2_ carbon dioxide, K^+^ potassium, Na^+^ sodium, Ca^++^ ionized calcium, Cl^-^ clorum, CK creatin kinase; LDH lactic dehydrogenases; ALT alanine-amino-transferase; AST aspartate-amino-transferase; DGGR 1,2-o-dilauryl-rac-glycero-3-glutaric acid-(6’-methylresorufin) ester; A/G albumin/globulin; P phosphorus; seroamiloid A; Na^+^/K^+^ sodium/potassium | | | | | | | | | | |
